# Corneal Subbasal Plexus in Eyes with Fuchs' Endothelial Corneal Dystrophy after Two Different Endothelial Surgeries

**DOI:** 10.1155/2021/5098272

**Published:** 2021-10-04

**Authors:** Irene Abicca, Marta Gilardi, Daniela Giannini, Rossella Anna Maria Colabelli Gisoldi, Augusto Pocobelli, Domenico Schiano Lomoriello

**Affiliations:** ^1^IRCCS–Fondazione Bietti, Rome, Italy; ^2^Rome Eye Hospital, Rome, Italy; ^3^San Giovanni Addolorata Hospital, UOC Oftalmologia-Banca degli occhi, Rome, Italy

## Abstract

**Purpose:**

To evaluate the morphological features and density of corneal subbasal plexus (SBP) using in vivo corneal confocal microscopy (IVCCM) in patients affected by Fuchs' endothelial corneal dystrophy (FECD) six months after Descemet membrane endothelial keratoplasty (DMEK) and Descemet-stripping automated endothelial keratoplasty (DSAEK).

**Methods:**

We included patients affected by FECD, requiring corneal endothelial surgery due to corneal oedema occurred from 3 to 6 months. 7 eyes underwent DMEK and 7 eyes DSAEK. All patients performed IVCCM preoperative and in six months postoperative. We analyzed SBP parameters, using CS4 Nerves Tracking Tool, and we studied the differences between the two endothelial keratoplasties.

**Results:**

Comparing the eyes treated with DMEK with those treated with DSAEK, preoperative corneal thickness, corrected distance visual acuity (CDVA), and age were similar in both groups. SBP was not detectable at preoperative IVCCM in any eye. Postoperatively, the nerve fibers length, the nerve fibers density, the tortuosity, and the number of fibers and of branching did not differ in the eyes that underwent DMEK compared to DSAEK. The corneal beadings density was higher after DMEK than DSAEK, and this difference was statistically significant (*P* = 0.004). The type of endothelial keratoplasty was not associated with the presence or absence of postoperative corneal SBP (Pearson' chi-square, 0.755).

**Conclusions:**

Postoperative corneal reinnervation should be easily and noninvasively studied using IVCCM. Morphological postoperative features of SBP did not differ between two different types of endothelial keratoplasty, DMEK and DSAEK, despite the different sizes of the corneal incision. The lower beading density in the DSAEK group should be the consequence of a different distribution of mitochondria along the nerve fibers, as expression of a supposed higher metabolic distress in the DSAEK group.

## 1. Background

Fuchs' endothelial corneal dystrophy (FECD) is a bilateral posterior corneal disease characterized by the loss of corneal endothelial cells and the development of posterior focal guttae, which are caused by Descemet membrane (DM) outgrowth [1]. Disruption of corneal endothelial pump-leak function can lead to corneal oedema and reduce visual acuity [2]. The FECD represents one of the most common indications for corneal transplantation worldwide [3, 4], and over the last two decades, significant surgical developments have been made for endothelial diseases. In particular, the full-thickness penetrating keratoplasty (PK) has been replaced by the posterior lamellar transplantation techniques: Descemet-stripping automated endothelial keratoplasty (DSAEK) and Descemet membrane endothelial keratoplasty (DMEK) [5, 6]. In the first technique, DSAEK, healthy donor endothelium with DM, and a variable thickness of posterior stroma is used to replace the diseased host endothelium [7]. Unlike the DSAEK, DMEK consists of the selective transplant of DM and endothelium [8–10].

The posterior lamellar techniques have several advantages compared with PK as lower incidence of intraoperative and postoperative complications, including rejection rates. Furthermore, regarding corneal innervation, endothelial keratoplasty is expected to have the ability to preserve fibers, while PK chopped off nerves both of the donor and of the host cornea [11–13].

The posterior lamellar surgeries also differ from each other in corneal incision size (4.1 mm and 2.8–3.0 in the DSAEK and DMEK, respectively) [7, 9], and this factor should influence the preservation and postoperative recovery of corneal nerves.

A rapid, noninvasive, high-resolution, and real-time imaging technique that can provide images of corneal fiber nerves is represented by in vivo corneal confocal microscopy (IVCCM) [14]. This examination allows, in particular, the analysis of the subbasal nerve plexus (SBP), placed between Bowman's layer and the basal epithelium in a radial distribution [15, 16]. Thus, IVCCM has demonstrated to be an important tool for studying the SBP after different corneal surgeries, as PK [12], laser assisted in situ keratomileusis (LASIK) [17], and DMEK [11].

Currently, in literature, there is any study comparing the SBP features between these two endothelial keratoplasties, DMEK and DSAEK. Consequently, we would investigate whether the choice of the surgical lamellar technique could also influence the corneal innervation in the follow-up of the surgery.

Therefore, the aim of our study is to evaluate the morphological features and density of SBP using IVCCM in patients affected by FECD at six months after DMEK and DSAEK.

## 2. Methods

### 2.1. Study Population

We enrolled patients affected by FECD requiring corneal endothelial surgery due to corneal oedema, who referred to the Anterior Segment Unit of IRCCS Fondazione Bietti, Rome, Italy, from November 2017 to May 2019. Patients with previous uneventful cataract surgery, performed more than 6 months before corneal surgery, were enrolled. We included the eyes with corneal oedema occurred for at least 3 and more than 6 months prior to keratoplasty. The onset of corneal oedema was evaluated by slit lamp microscopy at each preoperative visit. One eye for each patient was considered.

Exclusion criteria were as follows.

1. Presence of any corneal disease, as herpetic keratitis or stromal scar, and/or the history of previous refractive, glaucoma, or retinal surgery
2. Diagnosis of ocular disease which could influence visual outcome, as maculopathy, optic neuropathy, or amblyopia
3. History of diseases inducing a peripheral neuropathy (diabetes mellitus, inflammatory diseases, alcohol abuse, vitamin deficiency, malignancy treated with chemotherapy agents, chronic liver or renal failure, central nervous system diseases, entrapment mononeuropathies, and cervical or lumbosacral radiculopathies)
4. Intra or postoperative complications of corneal endothelial surgery

All patients underwent a complete ophthalmologic examination, such as corrected distance visual acuity (CDVA) (LogMar), slit-lamp biomicroscopy, intraocular pressure measurement using the Goldmann applanation tonometer, and fundus examination using the indirect ophthalmoscope before and 6 months after surgery. Endothelial cell count (ECD), corneal pachymetry, and SBP features were collected using IVCCM (ConfoScan 4; Nidek Technologies) before and after 6 months from surgeries.

All research procedures described in this work adhered to the tenets of the Declaration of Helsinki and were performed for clinical purposes using routine techniques. All recruited subjects gave written informed consent. The informed consent forms include consent for the use of anonymized instrumental results for scientific publications.

### 2.2. Surgical Procedures

Skilled surgeons (DSL and AP) performed the endothelial keratoplasty of the included patients as previously described [5, 7]. Specifically, the donor tissue was inserted through a corneal incision of 4.1 mm and 2.8–3.0 in the DSAEK and DMEK, respectively. The size of descemetorhexis was approximately 8.5–9.5 mm in both techniques [18].

The postoperative treatment was an association of topical antibiotics and prednisolone acetate 1%, administered 4 times a day, tapering to once daily by 2–6 months after surgery in all cases.

Intracameral air bubble or gas (20% sulfur hexafluoride, SF_6_) was used to facilitate tamponade of the graft to the host cornea. If it was necessary, the day after the operation anterior chamber was refilled by air/gas.

### 2.3. In Vivo Corneal Confocal Microscopy (IVCCM)

IVCCM (ConfoScan 4; Nidek Technologies, Gamagori, Japan) of the central cornea was performed in all patients with a z-ring adapter. All examinations were carried out by the same experienced operator (DSL). We applied to the tip of the lens a transparent and sterile gel (dexpanthenol 5%) to eliminate optical interfaces with different refractive indices, to keep constant the refractive index, and to allow a no-contact examination. After autoalignment, a full-thickness scan of the cornea was performed with 72% light intensity and a 6 *μ*m scan step, as previously described [19].

Corneal thickness was obtained measuring the distance between the endothelium and the last clear and the centred frame of epithelial image and ECD, using automated cells analysis of the central or paracentral area of the best image selected for the analysis [20].

### 2.4. Corneal Subbasal Nerve Plexus Analysis

Two experienced researchers (MG and IA) carefully examined only the images between the basal epithelial layer and Bowman's layer. They were masked to group assignment and cannot relate each image to the performed surgery. They selected the best focused frame of the SBP for each patient without motion folds and without more than one layer capture. The frames were analyzed using CS4 Nerves Tracking Tool CS4 software v1.3.0, each area was reviewed and any error was manually edited, after automated identification of fibers. Each operator (MG and IA) worked separately. In case of mismatch, a third operator (DG) chose the best option.

The corneal SBP parameters analyzed [21] were as follows.

1. Nerve fibers length (*μ*m/frame): the total length of all fibers and branches in a frame
2. Nerve fibers length density (*μ*m/mm^2^): the total density of the nerve fibers in mm^2^
3. Number of fibers: the total number of nerve fibers, including main nerves and branches
4. Number of branching: points where nerve branches arise from the main nerve
5. Nerve fiber tortuosity using Nidek Nerve index, a unitless measure which represents the degree of twistedness of a curved structure
6. Beadings: well-defined hyperreflective points along the corneal fiber, which are an agglomerate of mitochondria and glycogen. They consequently represent an expression of oxidative damage, and the study of their characteristics should help to evaluate the metabolic stress of corneal fiber [22]. We explored beadings features through two indices.

- Number of beadings: the total number of beadings identified in the main nerves (trunks, long fibers that crossed the borders of the area of analysis)
- Beadings density (beadings/mm): the total number of nerve beadings divided by the total length of nerve trunks in millimetres

We excluded patients showing no evidence of SBP in postoperative IVCCM.

### 2.5. Statistical Analysis

All the analyses were performed using Statistical Package for Social Sciences (SPSS), IBM Corp., Statistics, version 25.0. All results were expressed as the mean ± standard deviations. The normal data distribution was tested by using the one-sample Kolmogorov–Smirnov test. The independent sample *t*-test or the Mann–Whitney test was applied, as appropriate, to compare subbasal plexus parameters changes between DMEK and DSAEK groups. To study the relationship between corneal nerves parameters, the Spearman correlation coefficient was computed. In all analyses, *P* < 0.05 was considered to be statistically significant.

## 3. Results

Thirty-two eyes of 32 patients, affected by FECD and scheduled for an endothelial keratoplasty, performed IVCCM. Due to the persistence of corneal oedema and the presence of subepithelial haze, SBP was not detectable in a subgroup of patients (10 eyes of the DMEK group and 8 eyes of the DSAEK group), so we decided to exclude them.

Therefore, our study population included 14 eyes; 7 eyes (50% of 14 eyes) underwent DMEK, while the remaining 50% underwent DSAEK. Any patient underwent a postoperative anterior chamber rebubbling. The age was similar between the two groups (*P* = 0.55). Females were 85.71% and 71.43% in the DSAEK and DMEK groups, respectively.

No differences were found in preoperative corneal thickness between groups, 650.75 ± 81.21 and 662.5 ± 64.77 micron, respectively, in case of DMEK and DSAEK. Due to the presence of confluent guttae and diffuse oedema with subepithelial haze, preoperative ECD and preoperative SBP were both not detectable at IVCCM in any eye. Preoperative CDVA was 0.63 ± 0.24 and 0.64 ± 0.25 in case of DMEK and DSAEK, respectively (*P* = 0.970).

We analyzed the characteristics of SBP at IVCCM, using CS4 Nerves Tracking Tool, and we compared the DMEK and DSAEK groups at 6 months after surgery.

None of the postoperative ocular characteristics analyzed differs significantly between DMEK and DSAEK, as given in Table 1. Comparing the SBP parameters, only the corneal beadings density was higher after DMEK than DSAEK, and this difference was statistically significant (Table 2).

We found that, after DMEK, corneal nerve tortuosity of SBP showed a high direct correlation with postoperative thickness (*r* = 0.865; *P* = 0.012), and the same correlation was present between the number of branching and the age of patients (*r* = 0.817; *P* = 0.025).

In the DSAEK group, instead, no correlation was found between corneal nerve parameters and postoperative corneal thickness. Nevertheless, an inverse and high correlation was found between age and two corneal nerve parameters and specifically, the number of fibers (*r* = −0.837; *P* = 0.019) and the number of corneal beadings (*r* = −0.793; *P* = 0.033).

The type of endothelial keratoplasty was not associated to the presence or absence of postoperative corneal SBP (Pearson' chi-square, 0.755).

## 4. Discussion

This is the first study comparing postoperative corneal innervations in patients affected by FECD who underwent two different techniques of endothelial keratoplasty, DMEK and DSAEK.

Morphological alterations of SBP were already described in FECD. In particular, nerves density, length, and bifurcations should be lower than healthy corneas, even at early stages of disease [23, 24]. As the FECD got worse, the SBP nerves decreased, up to being completely absent in the severe stages [2].

FECD is one of the main corneal disease requiring a posterior keratoplasty, DSAEK and DMEK.

Preoperative and postoperative innervation in the eyes affected by FECD and treated with DMEK was speculated by Bucher et al., reporting a consistent reduction of number and length fibers in the early postoperative. Nevertheless, up to 4 months post-DMEK, a complete recovery of subbasal plexus was shown [11]. Similarly, after Descemet-stripping endothelial keratoplasty (DSEK), Ahuja et al. described regeneration of the subbasal nerve through 36 months, but with an irregular branching compared to healthy [23].

A small corneal incision at the limbus was necessary in both surgeries, DMEK and DSAEK, as cataract surgery. In literature, SBP alterations, even in uneventful cataract surgery, were already reported [25]. Authors suggested that, in endothelial keratoplasty, at first, the surgical trauma, and in particular, limbar incision and descemetorhexis, induced some fiber transection and a transient reduction of them. Subsequently, due to the release of neurotrophic factors by the graft cells, the nerve fibers could regenerate [9, 23].

Since the size of the corneal incision during DMEK was smaller than during DSAEK (2.8–3.0 versus 4.0 mm), we decided to investigate the postoperative characteristics of SBP after these two different posterior surgeries and to compare them.

We performed preoperative IVCCM and we did not identify any corneal subbasal fiber because of subepithelial haze associated to corneal oedema. However, as our purpose was to compare the postoperative SBP after the two different keratoplasty, we included corneas with similar preoperative characteristics (corneal thickness, age, and CDVA), but sufficiently transparent at 6 months after keratoplasty, and with low subepithelial haze to assess the characteristics of subbasal plexus.

We found that the corneal nerve fibers length, the nerve fibers density, the tortuosity, and the number of fibers and branching did not differ in patients who underwent DMEK compared to DSAEK.

The total nerve length and the number of branching recovery reported by Bucher [11] were higher than our postoperative data in the DMEK group. Unlike Bucher's group, our sample included the eyes without an evident preoperative SBP, due to subepithelial haze and diffuse oedema. We assumed that our corneas belonged to a more severe stage than those described by Bucher and that consequently, our postoperative recovery should require more time.

The most consistent reason for the corneal fiber injury after posterior surgery was, in our opinion, the surgical trauma and specifically, the corneal incision and descemetorhexis. Our results showed that the different sizes of corneal incision between DMEK and DSAEK seemed to not affect the main parameters of the corneal subbasal plexus. We also concluded that the type of endothelial surgery did not influence the presence of subbasal corneal plexus at 6 months postoperative (Pearson' chi-square, 0.755).

Our study group included patients with an average age of over sixty (66.26 ± 6.1 and 68.86 ± 9.41 years in the DMEK and DSAEK groups, respectively). Previously in literature, the lowering of corneal nerve density had been described in association with ageing [26, 27]. Our analysis was not affected by this age-related variability because in the two groups, patients treated with DMEK and those with DSAEK did not show a statistically significant difference. Age was highly related to some postoperative SBP features. In particular, branching was directly correlated to age in the DMEK group, and a negative correlation with the number of fibers was shown in the DSAEK group. Data in literature about relationship between age and corneal nerves parameters alterations were discordant, probably due to different methods applied, and it was beyond the scope of our study.

We examined the metabolic activity of corneal fibers through the analysis of beadings, which are an agglomerate of mitochondria and glycogen along the nerves [28].

We found that the number of corneal beadings was similar between the DMEK and DSAEK groups (*P* = 0.942), but the beading density was lower in the DSAEK group (*P* = 0.004). We described, for the first time, data regarding the corneal beadings after posterior lamellar corneal surgery. We supposed that these alterations should be the consequence of a different distribution of mitochondria along the nerve fibers, as expression of a supposed higher metabolic distress in the DSAEK group. The number of beadings per frame was similar between the two groups, but not the density of beadings along the total length of the trunk, probably due to the lower average length of the fibers after DMEK than after DSAEK, although not having a statistically significant difference. A preoperative analysis of beadings in patients affected by FECD should be useful to give clinical significance to our result. However, an interesting result of our study was that the beadings density at 6 months after DMEK was similar to the previously described, by our group, in healthy patients (71.09 ± 8.93 in our DMEK group versus 71.37 ± 10.30 in healthy corneas), showing indirectly a good metabolic balance of subbasal plexus at six months after surgery [21]. We concluded that the damage on the corneal fibers was similar between the two surgeries, but that the postoperative metabolic stress was greater in DSAEK than in DMEK. However, the main limitation of our study was the small number of corneas included. It was the first time that corneal beadings were analyzed after posterior keratoplasty, and these results will need to be studied on a larger population.

## 5. Conclusions

Corneal subbasal plexus did not show morphological postoperative differences between two different types of endothelial keratoplasty, DMEK and DSAEK. In the DSAEK group, we found a lower beading density, which should be the expression of a supposed higher metabolic distress in this group. IVCCM is a useful and noninvasive tool for the speculation of postoperative reinnervation.

## Figures and Tables

**Table 1:** Postoperative characteristics of study population.

	DMEK (*n* = 7) (mean ± SD)	DSAEK (*n* = 7) (mean ± SD)	*P* value
Age (years)	66.29 ± 6.1	68.86 ± 9.41	0.55
Postoperative ECD (cell/mm^2^)	1571.86 ± 355.44	1716.57 ± 583.71	0.142
Postoperative corneal thickness (*μ*m)	523.71 ± 48.28	583.71 ± 122.65	0.252
Postoperative CDVA (LogMAR)	0.03 ± 0.04	0.05 ± 0.04	0.304

**Table 2:** Summary of corneal nerves morphological parameters of study population.

Corneal nerves parameters	DMEK (*n* = 7)	DSAEK (*n* = 7)	*P* value
Nerve fibers length (*μ*m/frame)	487.93 ± 304.16	630.88 ± 408.34	0.472
Nerve fibers length density (*μ*m/mm^2^)	5490.51 ± 3422.59	7098.98 ± 4584.93	0.472
Number of fibers (*n*°)	3.14 ± 1.95	4 ± 2.58	0.497
Number of branching (*n*°)	0.71 ± 0.76	0.71 ± 1.5	0.43
Number of beadings (*n*°)	31.43 ± 20.58	30.57 ± 22.44	0.942
Beadings density (beadings/mm)	71.09 ± 8.93	49.62 ± 12.97	0.004∗
Nerve fiber tortuosity (*n*°)	5.12 ± 2.03	4.88 ± 2.87	0.857

^*∗*^Statistically significant (P < 0.05 )

## Data Availability

The datasets used and analyzed to support the findings of this study are available in Supplementary Materials.

## Supplementary Materials

Supplementary Materials Supplementary files include the data used and analyzed to support the findings of this study (Dataset.pdf).
