# Supplementary material for: Corneal Subbasal Plexus in Eyes with Fuchs' Endothelial Corneal Dystrophy after Two Different Endothelial Surgeries
Source: J Ophthalmol. 2021 Oct 4;2021:5098272. doi: 10.1155/2021/5098272 (PMC8505089; doi:10.1155/2021/5098272)
Supplement: Supplementary Materials — Supplementary files include the data used and analyzed to support the findings of this study (Dataset.pdf). [file 5098272.f1.pdf]

ID	SEX	Age	Surgery Type	Eye	Preoperative CDVA LogMar	Postoperative CDVA LogMar	Interface Reflectivity	Subepithelial Reflectivity	PSB	Nerve Fibers				BEADINGS		Tortuosity
										corneal fiber length (microm/frame)	corneal nerves fibers length density	number of fibers	bifurcation	number of beadings	beading density	tortuosity index
1	F	70	DMEK	od	0.70	0.04	176	55	yes	539.79	6074.05	5	1	34	69.68	8.73
2	F	73	DSAEK	os	0.5	0.15	61	58	no plexus							
3	F	51	DSAEK	os	0.4	0.04	71	63	yes	992.78	11171.29	7	4	70	70.51	9.44
4	M	69	DSAEK	os	0.5	0.04	63	64	no plexus							
5	M	72	DMEK	os	0.50	0.00	108	66	no plexus							
6	F	66	DMEK	os	0.40	0.00	96	67	no plexus							
7	F	71	DMEK	os	0.50	0.00	139	70	yes	995.59	11202.92	6	2	71	80.53	5.49
8	F	71	DMEK	os	1.00	0.00	152	72	yes	771.71	8683.65	4	1	43	72.1	4.77
9	F	58	DMEK	od	0.50	0.04	152	84	yes	223.88	2519.23	1	0	19	84.87	4
10	M	63	DMEK	os	0.70	0.00	157	87	no plexus							
11	F	67	DMEK	os	1.00	0.04	81	90	no plexus							
12	M	69	DSAEK	od	0.4	0	63	92	yes	267.89	3014.42	2	0	13	48.53	6.13
13	M	78	DSAEK	os	0.4	0.04	69	96	yes	163.13	1835.58	1	0	9	55.17	0
14	F	62	DSAEK	od	0.7	0.04	102	103	yes	1127.81	12690.76	7	0	46	40.79	4.24
15	F	74	DSAEK	os	0.4	0.1	125	116	yes	411.73	4632.95	3	1	13	34.43	6.07
16	F	73	DSAEK	os	1	0.1	134	116	yes	1047.13	11782.87	6	0	40	38.2	3.81
17	F	75	DSAEK	os	0.7	0	212	119	yes	405.68	4564.97	2	0	23	59.69	4.47
18	M	63	DMEK	od	1.00	0.00	164	120	yes	348.11	3917.11	2	0	22	63.2	6.46
19	F	59	DMEK	os	0.40	0.00	212	128	yes	149.19	1678.8	1	0	9	60.32	2.48
20	F	63	DMEK	os	0.70	0.10	174	128	no plexus							
21	F	85	DSAEK	od	1	0.15	113	129	no plexus							
22	F	72	DMEK	od	1.00	0.10	118	143	yes	387.27	4357.78	3	1	22	66.94	3.94
23	M	74	DMEK	os	0.30	0.04	161	145	no plexus							
24	F	77	DMEK	od	0.50	0.10	126	145	no plexus							
25	F	51	DMEK	od	0.40	0.00	185	146	no plexus							
26	F	84	DMEK	od	0.40	0.10	197	161	no plexus							
27	F	73	DSAEK	os	1	0.1	106	162	no plexus							
28	M	70	DSAEK	os	0.5	0.1	95	166	no plexus							
29	M	58	DSAEK	od	0.7	0.1	146	174	no plexus							
30	F	59	DSAEK	od	0.4	0.04	68	188	no plexus							
31	F	67	DMEK	os	0.7	0.04	171	194	no plexus							
32	F	73	DSAEK	od	1	0.15	116	239	no plexus							
